# Keeping an AI on the mental health of vulnerable populations: reflections on the potential for participatory injustice

**DOI:** 10.1007/s43681-024-00523-5

**Published:** 2024-08-20

**Authors:** Giorgia Pozzi, Michiel De Proost

**Affiliations:** 1 Delft University of Technology, Faculty of Technology, Policy and Management, Jaffalaan 5, Delft, 2628BX The Netherlands; 2 Ghent University, Faculty of Arts and Philosophy, Department of Philosophy and Moral Sciences, Blandijnberg 2, Gent, B-9000 Belgium

**Keywords:** Epistemic injustice, Participatory injustice, Conversational AI, Capability sensitive design, Ethics of AI, Epistemology of AI

## Abstract

Considering the overall shortage of therapists to meet the psychological needs of vulnerable populations, AI-based technologies are often seen as a possible remedy. Particularly smartphone apps or chatbots are increasingly used to offer mental health support, mostly through cognitive behavioral therapy. The assumption underlying the deployment of these systems is their ability to make mental health support accessible to generally underserved populations. Hence, this seems to be aligned with the fundamental biomedical principle of justice understood in its *distributive* meaning. However, considerations of the principle of justice in its *epistemic* significance are still in their infancy in the debates revolving around the ethical issues connected to the use of mental health chatbots. This paper aims to fill this research gap, focusing on a less familiar kind of harm that these systems can cause, namely the harm to users in their capacities as knowing subjects. More specifically, we frame our discussion in terms of one form of epistemic injustice that such practices are especially prone to bring about, i.e., *participatory injustice*. To make our theoretical analysis more graspable and to show its urgency, we discuss the case of a mental health Chatbot, Karim, deployed to deliver mental health support to Syrian refugees. This case substantiates our theoretical considerations and the epistemo-ethical concerns arising from the use of mental health applications among vulnerable populations. Finally, we argue that conceptualizing epistemic participation as a capability within the framework of Capability Sensitive Design can be a first step toward ameliorating the participatory injustice discussed in this paper.

## Introduction

Advances in artificial intelligence (AI) applications in biomedicine are increasingly considered promising for mental healthcare support [1]. With the launch of new large language models, such as GPT-4, this excitement is mounting. For instance, smartphones are believed to have the potential to aid researchers and therapists in comprehending, predicting, and intervening in human psychological phenomena by monitoring the mental states and actions of their users. One particularly promising resource in this regard is the smartphone psychotherapy chatbot – an artificially intelligent bot that provides cognitive behavior therapy to users, aiming to enhance their mental well-being [2]. Several benefits are commonly mentioned concerning the use of these chatbots for mental health issues, including their cost-effectiveness, widespread accessibility, and availability in various languages [3]. As a result, they are considered an ideal tool, particularly in regions where there is a scarcity of therapists who can communicate in the native language of individuals in need of mental healthcare.

Sedlakova and Trachsel have considered how the use of conversational artificial intelligence (CAI) tools raises challenging ethical questions related to issues of authenticity, autonomy, and expanding access for vulnerable populations [4]. The vulnerable groups that Sedlavoka and Trachsel highlight are the elderly, adolescents, and underdiagnosed people. However, other vulnerable populations^1^, such as refugees, who lack access to mental healthcare due to historical and cross-cultural treatment gaps, ought to be more central to the discussion of CAI [6]. As research indicates, there is a general paucity of literature and a lack of evidence available regarding the uptake of mHealth interventions among refugees and other vulnerable populations [7].

When specific attention is paid to such populations and the use of CAI, various ethical concerns come to light. Principles of biomedical and AI ethics, such as beneficence, non-maleficence, explainability, and justice, are applied in the literature as well [8, 9]. However, the latter ethical value has only been limitedly explored thus far [10]. As one of the few studies in AI ethics on this concept, Gabriel investigates the relationship between AI and principles of distributive justice [11]. However, the rationale of the “ideal theory” famous from John Rawls’ *A Theory of Justice* could be radically put into question as non-ideal societies with injustices have historically been the norm rather than the other way around [12]. Moreover, recent philosophical writing on the scope of justice has also drawn attention to forms of injustice that do not involve material redistribution, but rather the harms persons could suffer through failures of recognition and discrimination [13].

Our focus in this paper is on a less familiar kind of harm that CAI can cause in health care, namely the harm to individual human persons as knowers. Thus, we put forward an analysis of the epistemo-ethical impact of CAI on vulnerable populations through the lens of the analytic framework of *epistemic injustice* [14]. These generally underserved populations ought to be central to our discussion of the medical ethics of CAI. Our considerations aim to offer a novel perspective under which the fundamental biomedical principle of justice needs to be scrutinized in broader terms in the face of the role acquired by these systems in crucial practices, such as mental health support, to be widely delivered to vulnerable populations. Considerations of epistemo-ethical difficulties in mental health are not new [15–17], though little attention has been given to the digital context and the epistemic consequences of CAI for the therapist-patient interaction.

Miranda Fricker recognizes two main forms of epistemic injustice that are to be considered the building blocks of her framework: *testimonial* and *hermeneutical injustice*. In general terms, testimonial injustice occurs at the interpersonal level when a hearer attributes to a speaker a reduced level of credibility for epistemically invalid reasons (e.g., due to identity prejudices). Hermeneutical injustice is a more structural notion that aims to capture a wrong done to someone when, due to their marginalization, they do not have the conceptual resources to make sense of and express to others their social experience. Both testimonial and hermeneutical injustices could play significant roles in CAI for mental health care. For example, a patient might not feel understood and heard if a CAI cannot appropriately decipher their testimonial offerings. Moreover, hermeneutical difficulties can emerge if the experience the patient is trying to convey cannot be effectively subsumed under the conceptual categories available to a particular CAI, thus creating conceptual misalignments [18].^2^ However, in what follows, we frame our discussion in terms of one broad form of epistemic injustice that such practices are especially prone to, given the technology’s nascent status: *participatory injustice*. This injustice tracks one comprehensive category of epistemic encounters: engagement as participants in knowledge generation [20]. So understood, participatory injustice takes place among two or more interlocutors but is not restrained to purely testimonial interactions in which epistemic exchanges are limited to conveying and receiving information. Participatory injustice aims to capture a whole range of epistemic activities in which a knower is unaccounted for in their capacity to make hypotheses, contribute to the formation of knowledge, and acquire self-knowledge, among others [20]. This broader form of epistemic injustice is to be understood as a wrong anchored in the assumption that a person is not capable of making significant offerings in epistemically relevant situations. It is thus harmful because it unjustifiably downgrades a person’s epistemic agency.

The dialogical nature of psychotherapeutic encounters is not only aimed at a transfer of information between patient and psychotherapist [21]. The therapist’s role is also to accompany the patient through self-reflection and, ultimately, self-understanding, leading them to rethink and re-evaluate certain possibly detrimental beliefs and form new ones [3]. Thus considered, the range of epistemic activities associated with a therapeutic relationship is wide and requires the full and active participation of the patient. This should take place in an environment in which they feel acknowledged, taken seriously in their concerns, and capable of successfully engaging in relevant epistemic activities. Hence, the extent to which the epistemic participation of patients in this rich sense is possible through the use of mental health chatbots needs to be critically scrutinized.

In this article, we proceed as follows. Section 2 discusses a case of a mental health Chatbot, Karim, deployed to deliver mental health support to Syrian refugees. This case substantiates our theoretical considerations and the epistemo-ethical concerns brought about by the use of mental health applications among vulnerable populations. In Sect. 3, we introduce the theoretical framework of participatory injustice. In Sect. 4, we consider how conceptualizing epistemic participation as a capability to be accounted for through the framework of Capability Sensitive Design could lead to the mitigation of participatory injustice when it emerges in connection with the use of mental health chatbots among vulnerable populations. Finally, we discuss our contribution to ongoing discussions on the ethics and epistemology of mental health chatbots.

## AI-mediated mental health support for vulnerable populations: the Karim Chatbot

The risks of using chatbots in vulnerable populations have received limited attention in the ethics of AI literature. Scholars have devoted particular attention to the potential accountability gap created by such systems where a therapist is absent. For example, in the case that someone expresses suicidal ideation, the system lacks the capacity to react appropriately, as dramatic consequences of chatbot responses in such delicate situations have sadly shown [22]. It is unclear that a CAI can be trained to handle such a crisis situation, and even more unclear who should take responsibility if the CAI fails to mitigate this harm as well as a human could have. In another case, a company called Koko, provided emotional support chat services based on GPT-3 for 4000 people in distress without asking for consent. When users became aware of this unauthorized experiment, many felt betrayed. The division of responsibilities in such an experimental situation was once again ambiguous [23].

Apart from responsibility concerns, the main argument put forward in the literature is the need for greater efficiency: estimations suggest that for every 100,000 people worldwide, there are about four psychiatrists on average; that number is much lower in most low- and middle-income countries with about one psychiatrist for the same amount of people [24]. In the face of the overall shortage of therapists to meet the psychological needs of vulnerable populations, the hype surrounding AI-based technologies is often seen as a possible remedy as the quest to automate therapy could democratize access. Particularly, smartphone apps or chatbots are increasingly used to offer mental health support, mostly through cognitive behavior therapy (CBT). As Tekin points out, the enthusiasm revolving around the use of these systems can be interpreted to be based on three main promises that these chatbots seem to be able to uphold [3]. The first is that digital phenotyping allows early diagnoses and treatments, thus improving patients’ chances of early recovery (this is arguably also a good way for patients who do not recognize alarming symptoms themselves to become aware of them and seek support). The second is that they represent an alternative solution for people who do not feel comfortable seeking psychological support due to the stigma attached to it. Arguably, sharing intimate concerns with a chatbot instead of a human agent could decrease patients’ fear of being judged by their therapist. The third, more general promise is that access to psychotherapy is increased through the use of these technologies, supporting populations whose mental health needs would not be otherwise met.

In this paper, we focus particularly on the third promise mentioned, i.e., the fact that these systems are supposed to provide mental health support to populations whose mental health issues would otherwise remain unaddressed, thus enabling broader access to mental health support. Considering the case of a mental health chatbot introduced to provide mental support to Syrian refugees^3^, we build upon Tekin’s skeptical arguments regarding the efficacy of such technology by broadening the landscape of ethical and epistemological issues connected to it. In particular, we do so by elucidating how these systems can be used with the risk of bringing about an epistemic injustice, more specifically, a participatory injustice (a form of epistemic injustice) in Hookway’s sense [20]. We argue that it is not epistemically and ethically justified to try to resolve a problem in terms of distributive justice (i.e., the fact that human therapists are a scarce resource, particularly in the context of refugee mental health) at the cost of causing new issues in terms of epistemic injustice. Moreover, we show that chatbots such as Karim will likely disappoint the expectations created by the third promise mentioned since, as we will argue, it considerably impairs the epistemic participation of refugees in therapeutic communication.

In order to bridge the gap to participatory injustice, let us reconstruct some characteristics of the chatbot of interest. In March 2016, the Silicon Valley start-up X2AI (now Cass) launched “Karim,” a psychotherapy chatbot, to support Syrian refugees in Lebanon [25]. The chatbot uses natural language processing, a form of artificial intelligence (AI), to simulate human conversations in Arabic through existing communication channels such as SMS texts or Facebook Messenger. The chatbot was piloted only on 60 Syrians “mostly men and boys”. This is a strikingly small pilot for scaling up to a large and vulnerable population: there are over one million Syrian refugees in Lebanon. X2AI developed the pilot in partnership with “Field Innovation Team”, a non-profit specializing in technology in disaster recovery, and the so-called “Singularity University”, the Silicon Valley business incubator and consultancy service. In the report of the Field Innovation Team, it is mentioned that the chatbot encountered issues with translation because of the many variations in Arabic dialects. Instead of using standard Arabic and Google Translate, they hired Syrians to resolve translation issues to the Damascus (Levantine) dialect [26].

Karim is not explicitly marketed as a psychotherapeutic tool but rather as a “friend” [25]. Several issues can emerge against this background. One has to do with how these systems should be conceived of in the first place. The FDA recently relaxed regulations regarding how mental health chatbots can be sold as medically grounded devices. In fact, in the face of the mental health crisis brought about by the COVID-19 pandemic, what was previously conceived as a “wellness” application can now be rebranded as a proper medical intervention [27]. So, even if the line between the extent to which chatbots similar to Karim can be considered proper medical devices is quite blurry, they are *de facto* used to provide mental health support. In the case of Karim, this applies to particularly vulnerable populations whose mental health needs differ substantially from other, more privileged populations.

The latter point seems particularly relevant in the face of the fact that Karim has been developed as a version of Tess, a chatbot used in the USA to support people with an anxiety disorder or mild depression. While Tess serves as a therapeutic tool supplementing and not replacing a human-human psychotherapeutic relation, the use of Karim among refugee populations is unsupervised by trained professionals [28]. However, particularly for refugees who have most probably experienced traumatic or even life-threatening events, the presence of a human psychologist is even more crucial in order to be able to intervene in a situation of emergency.

Moreover, as with many other mental health support tools, Karim has not been subjected to empirical scrutiny [29]. The few empirical studies that tested mental health applications report positive results on patients’ mental health; however, if the tool is a supplement and not a replacement for the psychological support that human therapists can provide. It is largely recognized that using mental health apps in an unsupervised setting is quite controversial, and its effect and possible perils are untested [30].

While all these considerations are central in the analysis of the ethical impact of these systems, in this paper, we aim to elucidate a more subtle issue related to the effective possibility of epistemic participation that people using a chatbot like Karim have. To unveil the occurrence of epistemic injustices in refugee mental health through the use of chatbots similar to Karim it is important to consider that, as Tekin points out, sociocultural factors have an impact on mental health and illness [3]. That is to say, the imposition of Western criteria of how psychotherapy should work upon Middle Eastern populations with a different sociocultural background encodes an *identity bias* into the technology, thus excluding people who do not identify with the standards it follows. This can create fundamental difficulties in making one’s experience accessible to the technology. The possible mismatch between users’ experience and the concepts available to the systems can be seen as a first step toward epistemic injustices arising in connection with the use of these technologies among vulnerable populations. In fact, users’ possibility to properly engage with these tools can be constrained due to a gap between their lived experiences and the pre-determined options encoded into the system [19, 31, 32].

To support these claims, consider the use of chatbots in refugee support as described by an interviewee in Madianou: “All chatbots are about pushing information out. Even ‘Refugee Text’ is: ‘tell us your status and we’ll give you some information on that basis’. Maybe at best it’s tailored information, but it’s not a conversation. […] Participation is hard to do. It’s easy to push out information” [28, p. 858]. Hence, people’s possibility to participate in an epistemically meaningful communicative experience can be strongly impaired. As we argue in the next section, this paves the way for instances of participatory injustice to emerge.

On a similar note, Sedlakova and Trachsel argue that “the CAI as an algorithm-driven system is good in providing quantified data or factual information which are limited in range. This type of knowledge can be categorized as third-person knowledge that can inform patients about relationships, human mind, or psychological processes. However, this type of knowledge is insufficient to gain new self-understanding and constitute a therapeutic change.” [4, p. 9] Here, it becomes clear that therapeutic interactions are not limited to passing on and receiving information, that is, to testimonial exchanges in a restrictive sense of the term.^4^ In contrast, therapy entails epistemically richer interactions and activities in which understanding, self-understanding, hypothesizing, and critically analyzing are only some of the many relevant ones.

Against this background, our central aim in this article is to provide a theoretically informed analysis of these issues and make their ethical and epistemological consequences more explicit. In the following section, we spell out the notion of participatory injustice against the backdrop provided by the case of the chatbot Karim just discussed.

## Epistemic harm beyond testimony: toward participatory injustice

There are still precautions regarding the therapeutic possibilities of chatbots due to the preliminary nature and the early stage of research in this area. Moreover, it is unclear that Chatbots as technological artifacts can constitute a testifier since such technologies, unlike people, lack moral character and well-being. In the literature on social epistemology, an “anthropocentric view of testimony” is commonly held based on the presupposition that only persons can participate in the act of testimony because only humans, in principle, can qualify as testifiers [34].

Because of this fact, we want to focus on the early phases of knowledge production and possible related harms. In Fricker’s standard view of testimonial and hermeneutical injustice, knowledge transmissions, in the form of credibility deficit and interpretative obstacles, are central. However, there are many other core epistemic activities related to the generation of knowledge beyond giving testimonies and conceptual interpretation. Other kinds of epistemic injustice are thus possible beyond those focused on by Fricker. For instance, Dotson conceptualizes a preemptive self-censoring of the content and expression of speakers’ testimonies as “testimonial smothering” [35]. Especially in (digital) therapeutic conversations, epistemic subjects do not only contribute knowledge or opinion. Rather, they are trying to pursue new lines of inquiry.

Christopher Hookway was the first to make this point in his critical commentary of Fricker’s monograph, where he emphasized the central importance of cooperative epistemic endeavors and argued that there is a wide variety of types of participant contributions that lead to the success of cooperative epistemic pursuits. These contributions reach well beyond offering or seeking testimony. He introduced the concept of the participatory perspective in epistemic injustice to describe how knowers could be unfairly excluded from participating in non-testimonial epistemic practices such as critically questioning, understanding, and imagining (rather than a mere “informational perspective”). Hookway argues that a wide range of genuinely epistemic harms can take place when participation in inquiry is unjustifiably hampered. As Hookway puts it, “the resources we make use of in exercising our epistemic agency are richer and more varied than is often supposed. Someone may not be credited as sufficiently trustworthy as an ‘epistemic agent’, and this judgment may reflect identity prejudices, even if their evaluation as unreliable is not made in the context of a straightforward testimonial exchange” [20, p. 153].

The author offers the example of a teacher who, although willing to take students’ informational questions seriously in their role as students, does not give a student uptake when they ask a question that is intended as a contribution to the inquiry itself. What happens in such cases is that someone who wishes “to be recognized as a member of a community of people collaborating in the attempt to improve understanding or advance knowledge” fails to be so recognized [20, p. 155]. When not taken seriously as a legitimate participant in knowledge production, an epistemic agent can lose epistemic confidence, thus becoming too hesitant in their contributions. When one’s questions are ignored, one may develop a habit of silencing oneself, not asking relevant questions that might forward the investigation [20]. Hookway’s approach broadens the very concept of epistemic injustice in a helpful way and underscores that what is common to a wide range of cases of unfair epistemic treatment that falls under the category of epistemic injustice is the compromise of the epistemic agency of a marginalized group.

Based on the above-described case of Karim, one could imagine a similar scenario to the one in the classroom just described. The refugees were not treated as potential participants in discussions on the development of the application but just as testing subjects who could ask for and provide additional information. This could be based upon a stereotypical view of the value of refugees’ contributions to the debate. Due to prejudice, the company fails to respect the refugee as a potential contributor to the discussion (or participant in the discussion). The result is that the refugeesbecome epistemically disabled or experience what Medina describes as “epistemically disempowered” because the company fails to take their mental health questions seriously [36].

We argue that the situation of participatory injustice just described predominantly occurs as a consequence of two assumptions seemingly built into the design of the chatbot under scrutiny. The first has to do with a participatory prejudice that amounts to regarding the intended users of the system as *objects* and not as participants in the epistemic activities ensuing from the use of the chatbot [37]. Carel and Kidd consider this form of prejudice related to more general medical practices in which the role of patients in interactions with medical professionals is often restricted to reporting or confirming symptoms or anagraphic information, excluding a more substantial epistemic involvement. The consequences of forms of epistemic objectification have thus a considerable moral dimension. When patients are treated exclusively as the objects of epistemic inquiries, they are deprived of a “capacity essential to human value” [14, p. 44] as they are degraded to a condition in which they cannot make an active contribution to epistemic endeavors. The exclusion of patients’ active offerings can lead to a neglect of their personal experience of illness, the testimony of which is often crucial to appropriate medical care since it can provide information that cannot be inferred from patients through medical procedures in which they play a passive role [38]. These considerations can be transferred to the case of interest since the user interaction with the system does not leave space for the kind of “cooperative epistemic inquiry” that would be needed for a successful interaction geared toward mental health support [39, p. 316]. The second consideration pertains to assumptions related to the *trust* that end users can, indeed, participate in an epistemically substantial way. As Medina points out, participatory justice “involves being trusted in one’s overall epistemic competence and participatory skills, and not just as a possessor of knowledge but also as a *producer* of knowledge” (our emphasis) [40]. According to this view, the failure to design the CAI in such a way as to allow genuine epistemic participation of the user could underlie the failure to entrust them with the capacity of epistemic participation. Both assumptions are detrimental to users’ epistemic standing in the ways previously described.

Let us also point out that the latter observation has a bearing on whether epistemic subjects interacting with the chatbot can fulfill their role as epistemically autonomous agents. As Tanesini points out, epistemic objectification in Fricker’s sense, i.e., understood as being denied the possibility to convey knowledge and testimony, hampers the epistemic value of intellectual autonomy since epistemic agents are effectively constrained in their role of informants [41]. The same issue can arguably also occur under a broader definition of epistemic injustice as participatory injustice in the case under scrutiny. Being the person interacting with the CAI the object rather than the subject of the interaction, their possibility to be an active and autonomous enquirer toward the purpose of establishing a therapeutic exchange aiming at mental health support remains precluded to them.

## Mitigating participatory injustice through capability sensitive design

It is sometimes suggested that the remedy to problems of participatory injustice is the development of individual virtues [20]. Fricker proposes “virtuous listening” as a helpful corrective but partial solution to issues of epistemic injustice [14]. However, some scholars stress the need to go beyond the dyadic instances of epistemic injustice on which Fricker often focuses, aiming for more encompassing solutions [42]. Particularly in the context of systematic epistemic injustices such as the ones brought about by AI-based systems, it seems appropriate to explore principles for the cultivation of epistemically just technologies, as well as social and political institutions [43, 44]. In a similar vein, we believe that the more comprehensive approach of Value Sensitive Design (VSD), especially its development through the capabilities approach, i.e., Capability Sensitive Design as outlined by Jacobs [45], can support epistemically just CAI deployed in mental health. Let us first reconstruct some main characteristics of the VSD approach before we turn to Capability Sensitive Design.

### Value sensitive design

The need to couple ethical considerations with design choices ensues from the consideration that a system’s design can bring about a positive and/or negative change and that technological artifacts function as “agentive amplifiers” in that they can create possibilities that were previously unavailable to the agent [46, 47]. It is widely agreed upon in the current debate that technologies are not value-neutral but rather the product of choices encoded into a system’s design. Therefore, it is paramount to shape technological developments with shared moral values [48, 49]. The overall aim of the VSD framework is thus to translate core values into normative considerations, which are further concretized into precise design requirements that can be implemented.VSD’s methodology is tripartite as the design process is considered from three interrelated levels: a conceptual, an empirical, and a technical level of inquiry [50]. In an iterative process of moral investigation, this methodology aims at defining values to be translated into a technology’s design.This is done through the analysis of technical requirements that lead to the practical implementation of the intended values. Instead of a retrospective ethical investigation, VSD’s goal is to incorporate ethically and socially relevant considerations from the beginning of the design process, thus shaping the technical conditions for an ethically sound technological development [51].

However, scholars considering the application of VSD have pointed out limitations pertaining to this approach in its standard formulation [52]. For example, one problem highlighted is the identification of stakeholders [53], resulting in the central question of whose values should be effectively included in the design process in the first place. A second criticism indicates that the normative dimension often remains underdetermined in this approach. As Jacobs and Huldtgren point out: “VSD makes no explicit commitment to particular ethical theories” [52, p. 1]. Umbrello and van de Poel address this issue by linking it to a further relevant shortcoming that comes to light also once VSD is applied to AI technologies (such as machine learning systems), namely its lack of sensitivity for political and social contexts [54]. These authors propose a human rights framework as a possible solution in which a context analysis precedes the identification of relevant values. A further widely discussed approach that aims to ameliorate the issues briefly described is Capability Sensitive Design (CSD), which we elaborate on in the following.

### Capability sensitive design

CSD is a framework combining the method of VSD with the capability theory advanced by Martha Nussbaum, thus backing up VSD with a needed theoretical underpinning. Jacobs has recently considered the application of this framework to AI systems in well-being and health [45]. In this section, we build upon Jacobs’ work and argue that CSD can be useful in addressing the problem of participatory injustice in the mental health CAI application of interest in this paper. To achieve this goal, we proceed as follows. First, we explain why it can be fruitful to conceive of *epistemic participation* as a capability in the first place. In the second step, we show that seeing epistemic participation as a capability embedded in the context of CSD has two beneficial effects. The first is that it provides us with the theoretical tools needed to spot a participatory injustice since, as we have seen, these can occur in a rather subtle manner. Second, it provides the theoretical basis needed for designers to critically question whether a particular CAI could bring about these issues, thus anticipating possible problematic outcomes in terms of participatory injustice.

Introducing CSD and Nussbaum’s capability approach in detail goes way beyond the scope of this paper, so we just focus on a few key aspects. The primary aim of this approach is to design technologies that enhance and expand users’ fundamental capabilities. Nussbaum lists ten central capabilities,^5^ “(1) being able to live a normal length of lifespan; (2) having good health; (3) maintain bodily integrity; (4) being able to use the senses, imagination, and think; (5) having emotions and emotional attachments; (6) possess practical reason to form a conception of the good; (7) have social affiliations that are meaningful and respectful; (8) express concern for other species; (9) being able to play; and (10) have control over one’s material and political environment” [45]. The assumption underlying Nussbaum’s capabilities list is that every individual has a right to pursue a life worth living and, to this end, they should be able to exercise these basic capabilities [45].

Fricker explores the link between epistemic injustice and the capabilities approach and argues that epistemic contribution can be conceived of as a fundamental human capability, thus deserving to be included in Nussbaum’s capabilities list [56].^6^ More specifically, Fricker conceives of epistemic contribution as a “combined capability” following Nussbaum’s tripartite definition of different capabilities [56]. A combined capability is one that is developed and trained but that requires certain social conditions to be in place for it to effectively flourish. An example would be the capacity to express one’s sexuality. The internal capacity to do so can be developed by an individual. However, it can turn into concrete expression only as long as suitable external conditions are in place. For instance, in a situation of oppression and/or discrimination, a person’s capability of expressing their sexuality would not acquire the status of a combined capability since disruptive societal mechanisms would prevent them from effectively expressing this capability. In a similar vein, Fricker claims that wrongful exclusion or lack of credibility for unjustified reasons means that a person does not receive the social uptake needed to transform her innate ability to transmit knowledge, into a capability that she can successfully exercise.

In her analysis of epistemic contribution as a capability, Fricker focuses particularly on social reciprocity, insisting on the fact that we are not only epistemic receivers but also epistemic givers. Informational material and interpretative material are the two forms of epistemic giving that constitute the epistemic capabilities constrained in cases of testimonial and hermeneutical injustice [56]. In framing epistemic contribution as a capability in these terms, it is evident that Fricker’s approach remains at the level of receiving and conveying information. From an informational perspective, a person’s capability of contributing epistemically would be limited if she was, due to prejudicial considerations, deemed as an untrustworthy informant, for instance. However, the participatory perspective we are interested in goes beyond this more restrictive understanding of a subject’s epistemic contribution, and so does the capability ensuing from it. In the following, we frame epistemic participation as a capability, thus moving beyond an information perspective.

### Epistemic participation as a capability

One of Nussbaum’s listed capabilities is particularly noteworthy in relation to participatory injustice, i.e., *being able to use the senses*,* imagination*,* and think* [57]. As pointed out in Sect. 3, Hookway’s account of participatory injustice captures forms of epistemic injustice that exceed informational exchanges between two or more interlocutors. The conceptualization of this form of injustice aims to shed light on practices that unfairly limit the subject in their possibilities not only to share information and knowledge but to *create* knowledge or gain a deeper understanding, among others. For example, in a therapeutic relationship, a patient does not only need conditions in place for them to be able to pass on information to their therapist through testimony (in a descriptive fashion, e.g., subject X is experiencing anxious feelings) but to hypothesize, challenge, and possibly change her beliefs. There are thus richer epistemic activities that are crucial to a successful therapeutic relationship and do not necessarily involve transmitting and acquiring information. We maintain that these activities can be performed if users have the possibility to exercise the capability of *epistemic participation*. This encompasses the central epistemic activities mentioned and could be thus considered a subcategory of the more general ability of imagining and thinking, as recognized by Nussbaum.

We thus conceive of epistemic participation as a more encompassing combined capability that exceeds Fricker’s informational approach. In fact, epistemic participation requires trust in the fact that a subject is competent in their ability to ask pertinent questions, advance understanding of a certain subject matter through critical scrutiny, inquiring into a problem’s solution. The activities that these capabilities comprehend go beyond the informational ability of receiving and sharing information. Nevertheless, similarly to the capability of epistemic contribution envisaged by Fricker, epistemic participation requires appropriate development that can succeed through societal uptake. Recalling Hookway’s example in the classroom, a positive, unbiased disposition of a teacher with respect to the epistemic competencies of her students of being able to advance knowledge and understanding of a particular subject matter are necessary conditions for their capability of epistemic participation to flourish. Therefore, both an informational perspective and a participatory perspective, i.e., the one under scrutiny in this article, can be captured by a capability approach that highlights individuals’ epistemic agency and focuses on the external conditions that allow the subject to realize their capability.

Now that we have clarified in which sense we can conceive of epistemic participation as a human capability, we need to consider how CAI for mental health support can endanger or enhance it. Jacobs understands CSD as following the tripartite division of VSD in conceptual, empirical, and technical inquiry [45]. The steps of each investigation are not linear but rather entail a successive back and forth in which these investigations mutually inform one another in a process of ongoing re-assessment. For this reason, it is not possible to analyze these dimensions in a compartmentalized way. However, due to the limited scope of this paper, we cannot elaborate on each component of CSD, so let us advance some initial considerations pertinent to the conceptual investigation in relation to the case previously discussed.

As Jacobs argues, the goal of the conceptual investigation is threefold: identifying the capabilities connected to the technology under scrutiny, focusing on the stakeholders impacted by the technology, and determining relevant conversion factors. In the case analyzed, the capability that we are interested in investigating is epistemic participation as previously described, and the stakeholders involved are, very broadly, vulnerable populations receiving mental health support through a CAI application (such as in the case of Karim previously analyzed). Let us turn to some considerations related to conversion factors.

Conversion factors encompass the degree to which a person is able to transform a resource, in this case, an AI-based technology, into a capability (i.e., epistemic participation) [45]. For the CAI under scrutiny to support refugees’ mental health, we thus need to consider which factors would prevent them from using the technology to enhance their capability of epistemic participation. Against the background provided in the previous sessions, two main factors need to be scrutinized.

The first relates to assumptions built into the technology regarding the role that users can play in their interaction with the mental health support app. As previously pointed out in Sect. 3, the assumption that refugees are objects instead of subjects of mental health support comes to light in the case in which they are confronted with information generated by the system but do not get to participate effectively in an exchange in a more epistemically substantial way. Changing this assumption and considering users as subjects of an interaction geared toward mental health support is the first conversion factor we need to account for to enhance their capability of epistemic participation. Otherwise, the possibility of developing this capability risks remaining, *by design*, precluded to the user.

The second consideration has to do with contextualizing the use of these systems for a particular population. To transform the CAI into an exploitable resource, we need to consider cultural diversity as a paramount conversion factor. The fact that Karim is the follow-up version of an app developed and implemented in the USA for people with mild depression or anxiety (see Sect. 2) presupposes that the way in which psychotherapy is delivered in Western countries can be applied to a population with a completely different cultural background and mental health needs. Such an assumption can result in a built-in bias that imposes Western values onto a culturally different population [58]. This can lead, in turn, to ethically problematic issues related to unfairness and discriminatory outcomes connected to the use of the CAI under scrutiny. Context-sensitive considerations pertaining to the societal values, background knowledge, and expectations of these systems’ target population are thus paramount to designing for their active epistemic participation *through* the technology.

## Discussion

The main goal of this paper has been to expand on the ethical and epistemological assessment of the use of mental health chatbots among vulnerable populations. More specifically, we aimed to show that using these systems to mitigate issues of distributive injustice due to the scarcity and/or unavailability of human therapists can, as a downside, bring about less explicit but not less harmful forms of epistemic injustice.

Drawing on the case of the chatbot Karim used to provide mental health support to Syrian refugees, we showed that these systems could lay the ground for a particularly harmful form of epistemic injustice, i.e., participatory injustice. As we have argued, this relates to the fact that these systems’ users are fundamentally constrained in many crucial epistemic activities that we would otherwise consider central to successful therapeutic interactions. These amount to the possibility of gaining self-understanding, inquiring into one’s own mental health situation, modifying a set of disruptive beliefs while leaving space for new ones, hypothesizing, and critically questioning, among many others. Against the backdrop provided by our analysis, this paper’s contribution to ongoing discussions on the ethics and epistemology of mental health chatbots is threefold.

First, our analysis provides reasons why, to achieve an ethically sound use of mental health chatbots, we need to ensure that the users’ epistemic status as autonomous knowers and inquirers is not endangered, crucially, *through* the use of these systems. To this goal, the framework of participatory injustice was applied to a novel field of inquiry, i.e., mental health chatbots. Our considerations of epistemic injustice in this context should play a complementary role to efforts aimed at addressing the problem in terms of distributive (in)justice. With this work, we aimed to show that very well-funded and justifiable concerns to provide wide-ranged mental health support (a distributive issue) cannot be met at the cost of neglecting other harmful consequences ensuing from the participatory injustice these systems can bring about. Caution is thus necessary when it comes to assessing whether technologies such as CAI can indeed meet patients’ need for mental health support.

Second, we shed light on the ethical issues of using these technologies, specifically among vulnerable and generally underserved populations whose circumstances often remain under-researched in their specificity. The case of the chatbot Karim brings problems related to the effort of finding a technological solution to critical societal problems, such as refugees’ mental health, to the forefront. In particular, from our analysis, it emerges that the social background and contextual specificities that characterize different populations interacting with these technologies should receive timely attention. The erroneous assumption that the target population interacting with a CAI is homogeneous can lead to the imposition of dominant values and, ultimately, to discriminatory and unjust outcomes. Moreover, failing to adapt the design of CAI to the needs of the intended population can strongly constrain or even completely nullify the usefulness of these technologies in mental health support. If participatory injustices emerge, as we discussed in the case of Karim, it is worth considering whether patients’ mental health has any beneficial effect derived from the interaction with the CAI.

Third, this paper provided initial considerations on conceiving epistemic participation as a central human capability and how the Capability Sensitive Design framework can ameliorate issues of participatory injustice in CAI technologies. Thus, we provided insights into how to address the epistemological and ethical issues identified that can be encountered using these technologies, specifically among vulnerable populations. However, our analysis was limited to conceptual considerations. Further research is needed to consider how the capability of epistemic participation can be translated into appropriate norms and design requirements to be built into CAI technologies in support of the mental health needs of vulnerable populations. The initial considerations advanced in this paper hopefully show the relevance of this approach analyzed in connection with CAI and the risks that epistemic injustices, in general, and participatory injustice, in particular, represent for epistemic agents interacting with these technologies.

## Conclusion

In this paper, we analyzed a form of epistemic injustice, participatory injustice, in the context of CAI technologies deployed for mental health support. The case of Karim, a chatbot developed to provide mental health support to Syrian refugees, has been used to illustrate the nature of the injustice under scrutiny. The idea that an epistemic injustice can go beyond the informational level and have an influence on broader epistemic activities (such as critically questioning and contributing to a knowledge enterprise) is relatable to the role that patients should be able to take up when seeking mental health support. We showed that current CAI systems risk exposing patients to participatory injustices, particularly if deployed among vulnerable populations and in the absence of the supervision of human experts. Through the analysis of epistemic participation as a capability in the context of Capability Sensitive Design in relation to these technologies, we attempted to provide a first modest amelioratory step to mitigate participatory injustice. However, more research is needed to assess the epistemological and ethical challenges connected to these technologies. We hope that our work will draw more attention to the analysis of forms of epistemic injustice arising in connection with mental health chatbots and how to effectively counter those.

## Footnotes

1 We acknowledge that there is a great deal of vagueness in the definition of vulnerable populations. Refugees should not be considered a vulnerable group per se, as each individual should be evaluated based on his/her inherent and situational fragilities and needs [5].

2 Initial considerations on the potential for testimonial and hermeneutical injustice through the general use of mental health chatbots can be found also in this commentary of De Proost and Pozzi [19].

3 It should also be noted that there is a fine line between the definition of refugee, migrant, and asylum-seeker. This is important to consider because each immigration status causes different ethical concerns.

4 Admittedly, Fricker’s definition of testimony in her discussion of testimonial injustice is quite encompassing, being understood in the broadest sense of the transmission of knowledge [14, 33]. However, Hookway takes the argumentation a step further considering epistemic activities that do not necessarily rely on receiving or transmitting information.

5 Nussbaum’s approach that provides a finite list of human capabilities arguably applicable to any individual irrespective of societal, cultural etc. differences does not remain uncriticized in debates revolving around the capability approach. See, for example, Claassen for a critical assessment [55].

6 Fricker’s claim that this capability deserves an extra spot on Nussbaum’s list needs more substantiation than we could possibly offer in this paper if we were to argue that the capability of epistemic participation should be added as well. Probably less controversially, we think the latter can be subsumed under Nussbaum’s formulation of the capability to use the sense, imagination, and think. This is the case because we have a broader conception of epistemic participation in mind than Fricker’s informational view. We expand on this point later in the section.
